# Real-time monitoring shows substantial excess all-cause mortality during second wave of COVID-19 in Europe, October to December 2020

**DOI:** 10.2807/1560-7917.ES.2021.26.1.2002023

**Published:** 2021-01-14

**Authors:** Sarah K. Nørgaard, Lasse S. Vestergaard, Jens Nielsen, Lukas Richter, Daniela Schmid, Natalia Bustos, Toon Braye, Maria Athanasiadou, Theodore Lytras, Gleb Denissov, Tatjana Veideman, Oskari Luomala, Teemu Möttönen, Anne Fouillet, Céline Caserio-Schönemann, Matthias an der Heiden, Helmut Uphoff, Kassiani Gkolfinopoulou, Janos Bobvos, Anna Paldy, Naama Rotem, Irene Kornilenko, Lisa Domegan, Joan O’Donnell, Francesca De Donato, Matteo Scortichini, Patrick Hoffmann, Telma Velez, Kathleen England, Neville Calleja, Liselotte van Asten, Lenny Stoeldraijer, Richard A White, Trine H Paulsen, Susana P da Silva, Ana P Rodrigues, Petra Klepac, Metka Zaletel, Mario Fafangel, Amparo Larrauri, Inmaculada León, Ahmed Farah, Ilias Galanis, Christoph Junker, Damir Perisa, Mary Sinnathamby, Nick Andrews, Mark G O'Doherty, David Irwin, Sharon Kennedy, Jim McMenamin, Cornelia Adlhoch, Nick Bundle, Pasi Penttinen, Jukka Pukkila, Richard Pebody, Tyra G Krause, Kåre Mølbak

**Affiliations:** 1 EuroMOMO hub, Statens Serum Institut, Copenhagen, Denmark; 2 Austrian Agency for Health and Food Safety, Vienna, Austria; 3 Sciensano, Brussels, Belgium; 4 Ministry of Health, Nicosia, Cyprus; 5 European University Cyprus, Nicosia, Cyprus; 6 National Institute for Health Development, Tallinn, Estonia; 7 Finnish National Institute for Health and Welfare, Helsinki, Finland; 8 French Public Health Agency (Santé Publique France), Saint-Maurice, France; 9 Robert Koch Institute, Berlin, Germany; 10 Hessisches Landesprüfungs- und Untersuchungsamt im Gesundheitswesen, Dillenburg, Germany; 11 Hellenic Centre for Disease Control and Prevention, Athens, Greece; 12 National Public Health Center, Budapest, Hungary; 13 Health & Vital Statistics Sector, Central Bureau of Statistics, Jerusalem, Israel; 14 Health Service Executive - Health Protection Surveillance Centre, Dublin, Ireland; 15 Dipartimento Epidemiologia del SSR, Lazio - ASL Roma 1, Rome, Italy; 16 Health Directorate Luxembourg - Division de l'inspection sanitaire, Luxembourg, Luxembourg; 17 Directorate for Health Information and Research, Pieta, Malta; 18 National Institute for Public Health and the Environment (RIVM), Bilthoven, the Netherlands; 19 Statistics Netherlands, The Hague, the Netherlands; 20 Norwegian Institute of Public Health, Oslo, Norway; 21 Instituto Nacional de Saúde Doutor Ricardo Jorge, Lisboa, Portugal; 22 National Institute of Public Health, Ljubljana, Slovenia; 23 National Centre of Epidemiology, CIBER Epidemiología y Salud Pública (CIBERESP), Carlos III Health Institute, Madrid, Spain; 24 Public Health Agency of Sweden, Stockholm, Sweden; 25 Federal Statistical Office, Neuchâtel, Switzerland; 26 Federal Office of Public Health, Bern, Switzerland; 27 Public Health England, Colindale, United Kingdom of Great Britain and Northern Ireland; 28 Public Health Agency, Northern Ireland, United Kingdom of Great Britain and Northern Ireland; 29 Public Health Scotland, Glasgow, United Kingdom of Great Britain and Northern Ireland; 30 European Centre for Disease Prevention and Control, Solna, Sweden; 31 World Health Organization, Regional Office for Europe, Copenhagen, Denmark; 32 Department of Veterinary and Animal Science, Faculty of Health and Medical Science, University of Copenhagen, Copenhagen, Denmark

**Keywords:** all-cause mortality, COVID-19 pandemic, Europe, EuroMOMO

## Abstract

The European monitoring of excess mortality for public health action (EuroMOMO) network monitors weekly excess all-cause mortality in 27 European countries or subnational areas. During the first wave of the coronavirus disease (COVID-19) pandemic in Europe in spring 2020, several countries experienced extraordinarily high levels of excess mortality. Europe is currently seeing another upsurge in COVID-19 cases, and EuroMOMO is again witnessing a substantial excess all-cause mortality attributable to COVID-19.

The coronavirus disease (COVID-19) global pandemic is ongoing, with Europe and the Americas the current epicentres of transmission. As at 1 January, 2021, over 82 million cases and 1.8 million deaths have been reported globally [1,2]. In Europe alone, the number of cases reported has now passed 26 million, with over 580,000 laboratory-confirmed COVID-19 deaths [3,4]. As many COVID-19 cases and deaths may not be confirmed by virological testing, and as the pandemic causes a range of indirect and collateral harms, the officially reported figures of laboratory-confirmed COVID-19 cases and deaths represent only a part of the total disease, mortality and overall public health burden associated with the pandemic. Here we report some noteworthy estimates of a marked increase in excess all-cause mortality in Europe coinciding with a steep second wave of COVID-19 in many countries since September 2020.

## Excess all-cause mortality during the first COVID-19 pandemic wave in Europe

The framework and methodology of the EuroMOMO network has been described earlier. Briefly, participating countries obtain weekly data on the number of all-cause deaths from civil registers or other official sources in nearly real-time. The all-cause excess deaths are defined as the observed minus the expected numbers of deaths, and are estimated by participating countries or subnational areas (federal states/cities) using the EuroMOMO statistical algorithm [5]. The EuroMOMO hub compiles these outputs then performs a weekly secondary pooled analysis using an age-stratified method [6], which generates a timely estimate that determines whether there were more deaths than expected in any one week.

**Figure 1:**
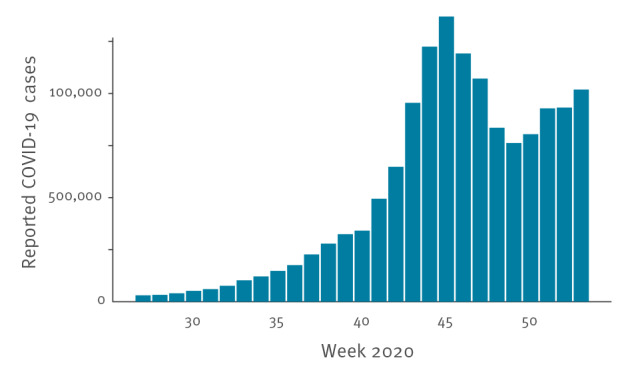
Weekly number of COVID-19 cases reported to the World Health Organization by the countries participating in the EuroMOMO network^a^, weeks 27 to 53, 2020

COVID-19: coronavirus disease; EuroMOMO: European monitoring of excess mortality for public health action; WHO: World Health Organization.

^a^ Detailed information about participating countries can be found on the EuroMOMO website [14].

The first wave of COVID-19 in spring 2020 was shown to be temporally associated with very high numbers of excess all-cause deaths in several individual European countries, as reported by the EuroMOMO network or its member countries [7-13]; most participating EuroMOMO countries implemented lockdowns during this time. During the summer period, COVID-19 case and death numbers were relatively low in general and social and physical distancing, as well as other public health measures, were relaxed in many countries [1]. In autumn 2020, the weekly number of COVID-19 cases again increased from approximately 341,000 total cases in week 40 (starting on 28 September) to 1.37 million cases in week 45 (starting on 2 November) in the countries participating in the EuroMOMO network (Figure 1).

Data updated on 7 January 2021. National data on COVID-19 cases reported to WHO represent the entire country, not just subnational areas, as is the case for the mortality data reported to EuroMOMO from some countries (e.g. Germany and Italy).

## Estimating excess all-cause mortality during second wave of COVID-19 in Europe

By the end of December 2020, the following 27 European countries or subnational areas had contributed their weekly mortality data: Austria, Belgium, Cyprus, Denmark, Estonia, Finland, France, Germany (Berlin and Hesse), Greece, Hungary, Ireland, Israel, Italy (19 cities), Luxembourg, Malta, the Netherlands, Norway, Portugal, Slovenia, Spain, Sweden, Switzerland and the United Kingdom (UK) (England, Northern Ireland, Scotland, Wales). Details of participating countries are available on the EuroMOMO website [14].

Weekly numbers of excess all-cause deaths were estimated for the total population (all ages) and for age groups 0–14, 15–44, 45–64, 65–74, 75–84 and ≥ 85 years. Furthermore, the number of weekly all-cause excess deaths was estimated from week 1 2020 up to and including week 53 2020, and compared with the mortality estimates from the previous 3 years (2017, 2018 and 2019), based on data received in week 1 2021. A country-specific adjustment function was applied to correct for the typical delay in registrations of deaths. Nonetheless, estimates of excess number of deaths for the most recent weeks are reported with some uncertainty and should be interpreted with caution (see more at [14]). Z-scores were applied to define and compare the excess mortality estimates.

### Excess mortality in the countries

During the second wave of COVID-19 transmission in the European Region, the all-cause mortality increased to a consistent and substantial level (exceeding 4 z-scores above baseline) initially in Israel around week 37 2020, followed by Spain in week 41, then by Italy (19 cities) and the Netherlands in week 42. Soon after, substantially increased excess mortality was also observed in Austria, Belgium, England, France, Greece, Hungary, Portugal, Slovenia and Switzerland. Denmark, Hesse (German federal state), Luxembourg, Scotland, Sweden and Wales have also observed some substantial excess mortality, but so far only as spikes. Meanwhile, several other countries or subnational areas had, by week 53, seen no or only limited excess mortality: Cyprus, Estonia, Finland, Berlin (German federal state), Ireland, Malta, Northern Ireland and Norway.

### Pooled excess mortality for participating countries

**Figure 2:**
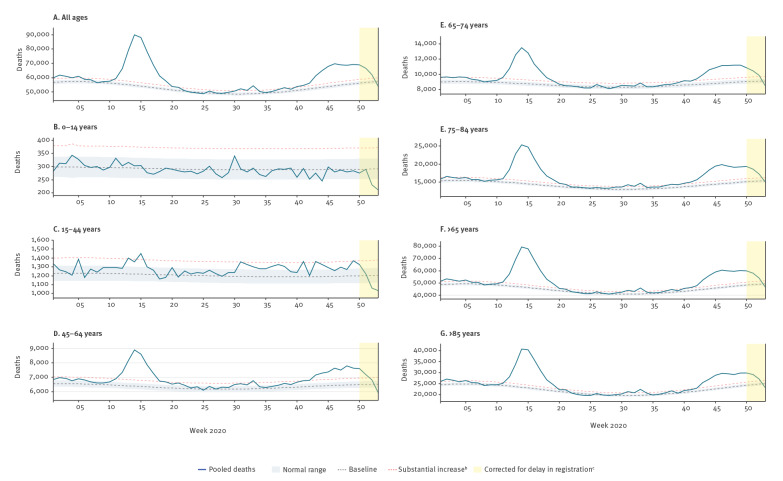
EuroMOMO pooled estimates of all-cause mortality (A) for all ages and (B–G) by age group, 27 participating countries^a^ or subnational areas, weeks 1 to 53 2020

The pooled estimates of all-cause deaths for the 27 participating European countries or subnational areas during the second wave of COVID-19 show an overall consistent and substantial increase in excess all-cause mortality from week 40 2020 (Figure 2), though there was a spike in week 38 2020. Excess mortality was highest among individuals aged ≥ 65 years, but we also observed substantial excess deaths among individuals aged 45–64 years. Low levels of increased excess mortality were seen in younger adults aged 15–44 years, while no excess mortality was seen in children aged 0–14 years.

EuroMOMO: European monitoring of excess mortality for public health action.

^a^ Detailed information about participating countries can be found on the EuroMOMO website [14].

^b^ Substantial excess mortality is defined as an excess of four z-scores or higher than the expected baseline level.

^c^ The last 3 weeks are marked in yellow, as there is some uncertainty about the estimates (as they were adjusted for typical delays in registrations of deaths).

Data updated on 7 January 2021. Please note the different scales of the y axes.

**Figure 3:**
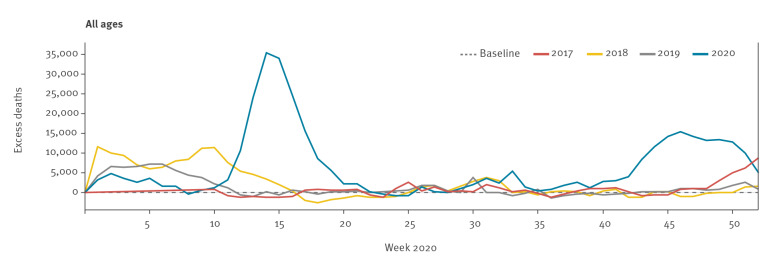
EuroMOMO pooled estimates of excess all-cause mortality for all ages by week and year, 27 participating countries^a^ or subnational areas, weeks 1 to 52 2017–2020

As at week 1 2021, our pooled estimates showed for the second COVID-19 wave a peak number of 15,392 excess all-cause deaths for all ages in week 46, corresponding to a z-score of 24.8. Although this is very high, this excess mortality level is still lower than the peak reached during the first wave of COVID-19 in week 14 2020, when the pooled excess mortality reached 35,408 deaths for all ages (z-score = 55), as seen in Figure 3.

EuroMOMO: European monitoring of excess mortality for public health action.

^a^ Detailed information about participating countries can be found on the EuroMOMO website [14].

Data was updated on 7 January 2021.

## Ethical statement

Ethical approval was not needed for the study, which is based on surveillance data only.

## Discussion

Excess all-cause mortality is widely recognised as a robust and comparable proxy for the total COVID-19–associated mortality in the population [15]. Based on long-standing experience of timely monitoring of weekly excess all-cause mortality across many European countries, the EuroMOMO network was able to promptly detect, quantify and report the full mortality impact associated with COVID-19 during the first wave in early 2020 [16].

The rapid increase in excess all-cause deaths seen in autumn 2020 was unusual for that time of year. Such a pattern of excess mortality is usually seen later in the autumn and during winter, and is associated with the seasonal transmission of influenza virus. The last time an early excess mortality was seen was in 2009, associated with the 2009 influenza A(H1N9) pandemic.

Similarly to spring 2020, the increase in excess deaths in Europe in the second wave of the COVID-19 pandemic has been observed in the age groups 45–64 years, 65–74 years, 75–84 years and ≥ 85 years. Nevertheless, some excess mortality has also been seen in individuals aged 15–44 years, but to a much smaller extent than among the older age groups. No excess mortality has been observed in children 0–14 years.

The estimates presented here represent excess all-cause deaths. As pointed out previously, in the absence of any major public health events other than the steep increase in reported COVID-19 cases, and with very low levels of seasonal influenza in the participating countries at present, the estimated excess mortality can be primarily attributed to the impact of the COVID-19 pandemic, either directly or indirectly [15]. Although our mortality estimates for weeks 51, 52 and 53 are uncertain and should be interpreted with caution, as our applied adjustment for the typical delay in registrations may be imprecise, we conclude that there was a consistent and substantial increase in excess mortality from week 40 2020 onwards. The delay in mortality following infection might even increase the observed mortality over the next few weeks.

## Conclusions

Our findings are a warning signal of the serious impact the evolving second wave of COVID-19 could have on mortality, as seen in many European countries. With a maximum z-score of 24.8, pooled mortality has not yet reached the same level as observed during the peak of the first wave in spring 2020 (maximum z-score 55) and COVID-19 transmission has seemed to stabilise in some European countries, associated with strengthening of control measures. However, it is important to note that mortality increases lag increases in cases. Indeed, the mortality level observed during the second wave of COVID-19 is already higher than the mortality peak seen during the severe influenza season in Europe in 2017/18. There is a strong need for continued attention and public health action to avoid a worsening situation. The recent commencement of a rolling programme of targeted vaccination against COVID-19 offers the prospect of significant blunting of excess all-cause mortality in 2021.
